# Diffusion model predicts the geometry of actin cytoskeleton from cell morphology

**DOI:** 10.1371/journal.pcbi.1012312

**Published:** 2024-08-05

**Authors:** Honghan Li, Shiyou Liu, Shinji Deguchi, Daiki Matsunaga

**Affiliations:** 1 Division of Bioengineering, Graduate School of Engineering Science, Osaka University, Osaka, Japan; 2 School of Life Science, Peking University, Beijing, China; Georg-August-Universitat Gottingen, GERMANY

## Abstract

Cells exhibit various morphological characteristics due to their physiological activities, and changes in cell morphology are inherently accompanied by the assembly and disassembly of the actin cytoskeleton. Stress fibers are a prominent component of the actin-based intracellular structure and are highly involved in numerous physiological processes, e.g., mechanotransduction and maintenance of cell morphology. Although it is widely accepted that variations in cell morphology interact with the distribution and localization of stress fibers, it remains unclear if there are underlying geometric principles between the cell morphology and actin cytoskeleton. Here, we present a machine learning system that uses the diffusion model to convert the cell shape to the distribution and alignment of stress fibers. By training with corresponding cell shape and stress fibers datasets, our system learns the conversion to generate the stress fiber images from its corresponding cell shape. The predicted stress fiber distribution agrees well with the experimental data. With this conversion relation, our system allows for performing virtual experiments that provide a visual map showing the probability of stress fiber distribution from the virtual cell shape. Our system potentially provides a powerful approach to seek further hidden geometric principles regarding how the configuration of subcellular structures is determined by the boundary of the cell structure; for example, we found that the stress fibers of cells with small aspect ratios tend to localize at the cell edge while cells with large aspect ratios have homogenous distributions.

## Author summary

We proposed a diffusion model-based machine learning system trained with cell outline and corresponding actin stress fibers data. The overall process can be divided into two steps. First, we cultured human fibroblast (HFF-1) cells and segmented cell outlines/stress fibers from microscopic images using a CNN (convolutional neural network) method and image processing techniques. Second, we trained the machine learning system with the diffusion model using sets of two corresponding images, the segmented actin stress fibers and the input images (cell outline). After adequate training, the network can transform from cell contour to its potential actin stress fibers distribution. This system can be well utilized for conducting virtual experiments predicting the distribution and localization of actin stress fibers by adjusting the input cell geometry. This system could provide a novel and experiment-free approach to quantify the stress fibers dynamics associated with the cellular morphology.

## Introduction

Actin stress fibers (actin SFs) are a prominent component of the actin-based intracellular structures and are mainly composed of actin and myosin [1], which generate strong contractile forces and play a vital role in cell motility and morphological changes [2]. SFs exhibit adaptive responses to changes in intracellular and extracellular cues by modulating their assembly and disassembly [3], which allow them to be involved in diverse cellular functions such as the regulation of cell–substrate adhesions [4, 5], cell migration [6], mechanotransduction [7, 8], morphological maintenance [9, 10], senescence [11], mechanical properties [12], and avoidance of proinflammatory signaling and maintenance of cell homeostasis [13, 14].

Cells sense mechanical properties in their surrounding microenvironment [15, 16] and change their geometric morphologies, both internal cytoskeletons and outer shapes, to modulate their functions. Many previous studies reported the geometric relation between the cell outline and the alignment/localizations of SFs. SFs, or actin filament bundles, are known to align along the longest axis of the cell shape (confinement), and this tendency becomes stronger for cells with larger aspect ratios [17–20]. The curvature of the cell contour, which can be obtained by fitting the cell outline with circles, also affects the localization of SFs and the contractile force as a consequence; SFs tend to be more concentrated where the local curvature is concave [21], and they are thicker at a place where the curvature radius is larger [22]. The curvature was previously used to estimate the contractile force of the cell [23, 24] since the tension of these SFs is in balance with the membrane tension [25], and this local curvature affects various cell functions such as differentiation [26]. Based on these experimental observations, some previous studies attempted to build simple models to predict the geometry and localizations of SFs out of limited information. Using geometric constraints of micro-patterning, previous studies [24, 27–29] have successfully described the localization of SFs and/or focal adhesions inside cells using one- or two-dimensional bio-chemo-mechanical models. More recently, there has been an attempt to predict the alignment of the actin cytoskeleton by modeling the cytoskeleton as a nematic liquid crystal [30]. In this model, they coupled the dynamics of cellular contour and the internal topologies, and discussed how these interplays impact the internal/outline geometries of cells. To summarize, while the previous studies provided vital understanding into the local characteristics of SFs, predicting the distribution of SFs across various cell shapes remains a challenge.

Although it is of interest to understand how the cell shape and cytoskeleton crosstalk in order to adapt to their microenvironments, it is still difficult to draw underlying geometric principles between them from the big data of experiments. Here, we present a machine learning-based system that predicts the geometry and localization of SFs from cell shape. To prepare training data, we extract cell shapes from microscope images using image processing methods and segment SFs using a convolutional neural network (CNN) method. Our machine learning system, which is based on the “diffusion model” [31], is trained to learn the rules to convert the input cell shape images to new images, predicting where SFs are likely to be in the corresponding cell shape. The diffusion model can generate the expected data distribution from random noise according to a given constraint. Note that we utilize the generative model instead of the simple CNN since CNN is unsuitable for generating geometries with sharp interfaces because of the nature of their loss functions: the loss function tends to average out pixel values, leading to blurriness. Although other generative models such as GAN [32–34] can be utilized for the present task, we used the diffusion model due to its training stability and the ease of hyperparameter tuning. We show that our system can recover important geometric features of SFs only from the cell shapes. Additionally, we utilize these geometric features to associate the local geometric features of cells (concavity and convexity) with their contractility, which provides possibilities for inferring mechanical information from the cell geometries. We also propose that this system can be used to perform virtual experiments, generating SFs localization maps from artificial cell shapes. The virtual experiments enable us to quantitatively assess the geometry of SFs under specific conditions, facilitating the discovery of underlying geometric principles that connect cell shape and SFs.

## Materials and methods

**Fig 1:**
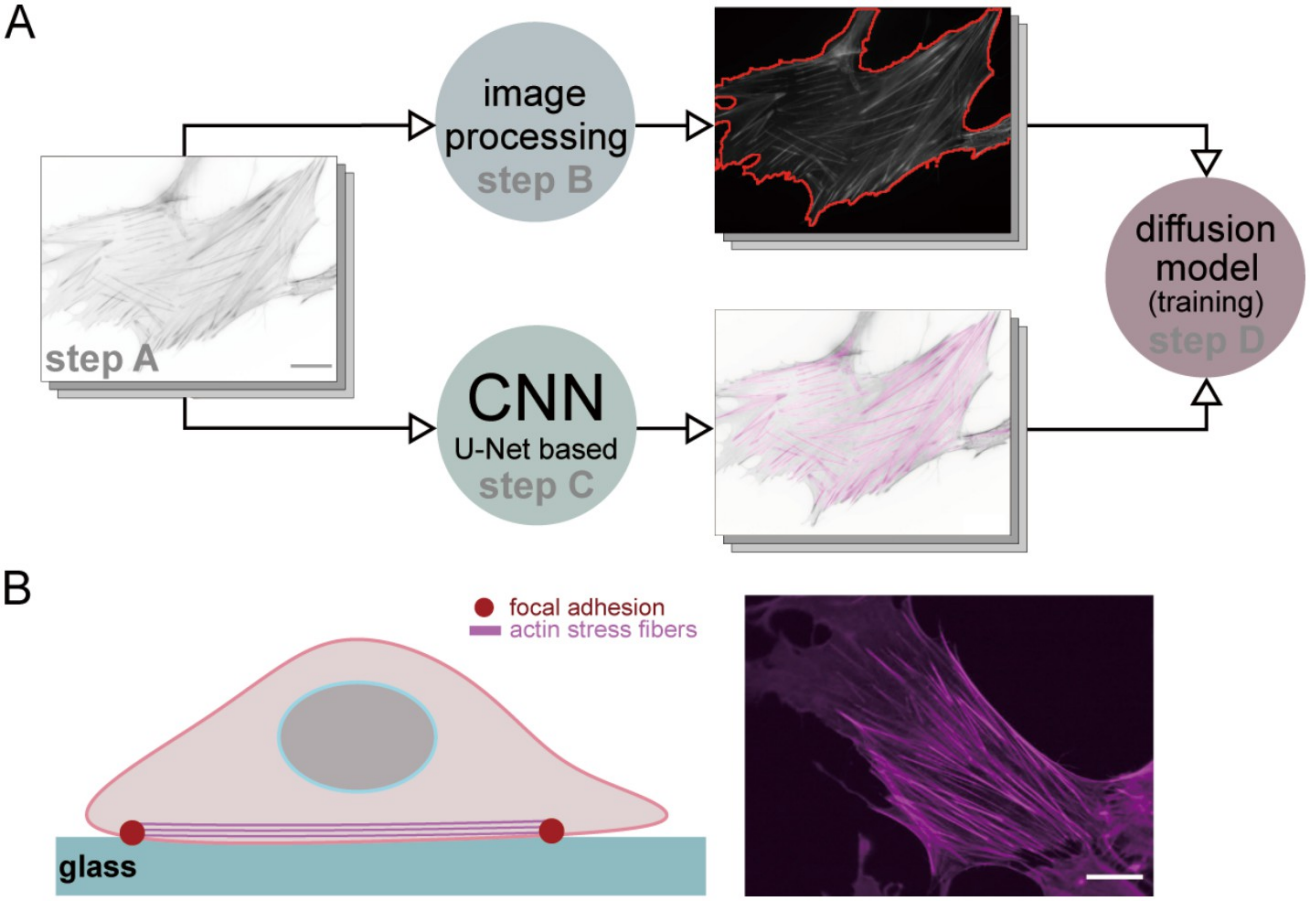
Overview of our approach and experimental material. (A) The outline of our method. By applying image processing techniques and the CNN method, the cell outline and SFs can be extracted from the original microscopic images. The extracted outline and SFs will be sent into the diffusion model so that the network can understand the transformation from cell outlines to SFs. (B) Schematic of cell culture. The cells are cultured on the glass substrate, and the SFs can be visualized by fluorescently-labeled phalloidin. Scale bars are 20 *μ*m.

Fig 1A summarizes our machine learning-based system. The process has four stages: (A) culture cells and acquire the cell images, along with the SFs, (B) extract cell outlines using a sequence of image processing methods, (C) segment the SFs using a CNN method, and (D) train the diffusion model using the SFs and cell shape so that the system understands the correlations between them.

### Step A: Experimental material

In this study, we utilized human foreskin fibroblasts HFF-1 (ATCC) to investigate the structure and dynamics of actin SFs. The cells were cultured in DMEM with high glucose, l-glutamine, and phenol red (Wako), which was supplemented with 15% fetal bovine serum (Sigma-Aldrich) and 1% penicillin-streptomycin solution (Wako). The culturing process was carried out in a 5% CO_2_ incubator maintained at 37°C. Before the imaging, the cells were fixed with 4% paraformaldehyde in PBS (Wako) at 37°C for 30 minutes, and permeabilized for 15 minutes using 0.1% Triton X-100. The actin SFs within the cells were stained using fluorescently labeled phalloidin (Thermo Fischer Scientific) as shown in Fig 1B. In total, 1293 images were captured using a confocal laser scanning microscope (FV1000; Olympus), equipped with a UPlan Apo 60× oil objective lens (NA = 1.42).

### Step B: Cell shape extraction

The details of the cell outlines are not sensitive to the image resolution; therefore, all the procedures in the cell outline extraction are executed under the resolution of 256 × 256, which is downsampled from 1024 × 1024. The whole process of the image processing algorithm for the cell outline extraction can be summarized as three basic steps. First, a Gaussian filter is applied to the raw microscopic images to remove the noise and the excessive local gradients caused by SFs, and the kernel size for the Gaussian filter is set to 8. Second, a binarization method with adaptive threshold [35] is applied to separate the cell outline from the background. Third, we applied morphological operations (erosion for two times and dilation two times) to the binarized images, which allowed holes and localized small connected components caused by the binarization to be removed from the images.

### Step C: Actin SFs segmentation

**Fig 2:**
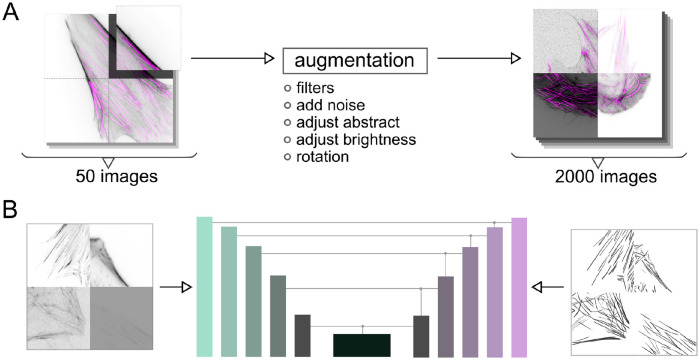
CNN method for the SFs segmentation. (A) Preparation of training data. By utilizing the augmentation methods, the original 50 images are increased to 2000 for the CNN network to be trained. (B) The CNN network (U^2^-Net) is trained with the augmented SFs data. After sufficient training, the network can automatically segment the SFs from microscopic images.

We proposed a CNN-based method to automatically segment the SFs from the raw fluorescence microscopic images. First, we prepared 50 original images with a resolution of 1024 × 1024 for the training data and 10 for the test data. The SFs are labeled by tracing the SFs with freehand lines, and SFs are labeled as 1 while the background is 0. Since this process requires high labor costs and time spent, we propose an image augmentation method to maximize the use of information from the labeled images. Since CNN is usually adept at capturing localized textural information [36], we cut each of the original 50 images into four pieces with equal length and width and combined them randomly, as shown in Fig 2A. During the combination, we utilized several image processing methods on each piece to simulate the data with unpredictable random noise that is introduced in experiments and data acquisition: (i) apply Gaussian filter and mean filter to imitate the out-of-focus state of the microscope, (ii) add noise to simulate the conditions with high background noise, (iii) randomly adjust the contrast and brightness of the images to reduce the impact of exposure intensity for different samples in the experiment during the training, and (iv) apply random clockwise rotation and flipping on the images to increase the diversity of training data. After these procedures, the total number of the training data augmented to 2000 from 50 original microscopic images.

Next, we built a CNN network to segment the SFs, as shown in Fig 2B. The architecture of this network basically refers to U^2^-Net [37], and we deepened the network so that it can process the input augmented SF data at a resolution of 512×512. We train the network by minimizing the differentiation (binary cross entropy) between the output and ground truth from the training data. We use the training parameters as follows: 50 training epochs (batch size = 4), the parameters *β*_1_ = 0.5 and *β*_2_ = 0.9 are used for the Adam optimizer, *ε* = 2.0 × 10^−4^ learning rate for the optimizer. Nvidia RTX A4000 and Nvidia Titan RTX accelerate the whole training process. After sufficient training, the CNN network could be utilized to segment the SFs.

### Step D: Training with diffusion model

**Fig 3:**
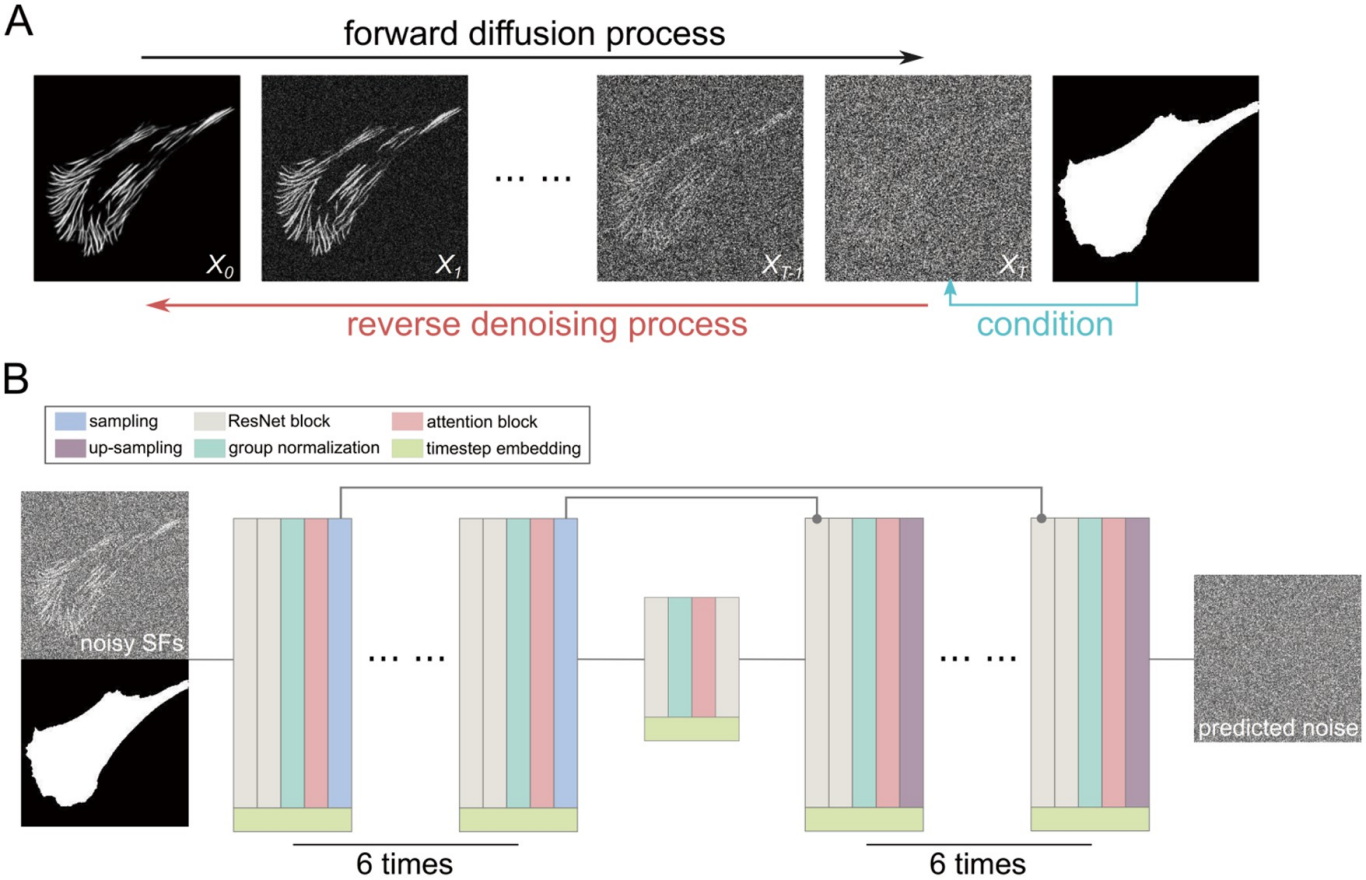
Generating SFs from cell shapes using the diffusion model. (A) Schematic of the diffusion model showing the forward process of turning SF images into Gaussian noise and the reverse denoising process of transforming Gaussian noise into SF images under a certain cell contour condition. (B) Architecture of the neural network used to predict noise added to SF data in the forward diffusion process under the cell contour condition. After training, the network can generate SF data from random noise based on the input cell contour.

The diffusion model [31, 38] mainly consists of forward diffusion and reverse denoising processes, as shown in Fig 3A. The forward diffusion process can be considered a Markov chain aiming to gradually add Gaussian noise to the input SF data until it becomes pure Gaussian noise after a total of *T* timesteps. The reverse denoising process is designed to recover the original SF data from the corrupted data produced by the forward diffusion process. By combining the reverse denoising process with the corresponding cell contour condition, it is possible to remove the noise from the data and recover the original SF data. This process is also illustrated in Fig 3A.

The forward diffusion process [31] can be expressed as follows,

$$\begin{array}{c} x_{t}=\sqrt{\bar{\alpha_{t}}}x_{0}+\sqrt{1-\bar{\alpha_{t}}}\epsilon \end{array}$$ (1)

where *x*_0_ are the initial input SF images, $\bar{\alpha_{t}}=\prod_{t}^{i}\alpha_{i}$ is the variance schedule, and *ϵ* ∼ *N*(0, 1) is the Gaussian noise. Note that *α*_*i*_ is an incremental sequence of values such that 0 < *α*_*T*_ < … < *α*_1_ < 1. While the reverse denoising noise process aims to gradually recover the corrupted *x*_*t*_ to *x*_0_ by estimating the noise in iterations [1, …., *T*]. This process can be modeled [39] as

$$\begin{array}{c} x_{\tau_{i-1}}=\sqrt{\alpha_{\tau_{i-1}}}\left(\frac{x_{\tau_{i}}-\sqrt{1-\alpha_{\tau_{i}}}\mu_{\theta }\left(x_{\tau_{i}},c,\tau_{i}\right)}{\sqrt{\alpha_{\tau_{i}}}}\right)+\sqrt{1-\alpha_{\tau_{i-1}}}\mu_{\theta }\left(x_{\tau_{i}},c,\tau_{i}\right) \end{array}$$ (2)

where *τ* is an increasing sub-sequence of [1, …., *T*] with length *S* for accelerating the reverse process [39], *c* denotes cell contour condition, *μ*_*θ*_ denotes the neural network, *θ* represents the learnable parameters in the network.

The objective of the neural network is to predict the Gaussian noise *ϵ* that is added in the forward diffusion process based on the input noisy SF images *x*_*t*_, contour condition *c* and the specific timestep *t*. The objective function of this process can be simplified to:

$$\begin{array}{c} L_{t}={∥\epsilon -\mu_{\theta }\left(\sqrt{\bar{\alpha_{t}}}x_{0}+\sqrt{1-\bar{\alpha_{t}}}\epsilon ,c,t\right)∥}^{2} \end{array}$$ (3)

where the neural network tries to minimize *L*_*t*_. The topology of our network, aiming to predict the noise distribution from input SF images added with different scales of noise, is designed refer to U-Net [40] with a contracting path, a bottleneck and an expansive path as shown in Fig 3B. In the contracting path, we designed a module that consists of several process units, which are ResNet block [41] for 2 times, group normalization [42], attention block [43] and sampling operation. While in the expansive path, we use the same module but replace the sampling operation with an up-sampling operation. The bottleneck path has successive modules of ResNet block, group normalization, attention block and ResNet block. Each module in the contracting path and expansive path repeats 6 times, and the output of each module in the contracting path is added to the input of corresponding modules in the expansive path. Each block has an additional concatenation with timestep embedding [44] in order to let the network know the current timestep.

In summary, the whole process of our diffusion model is to train a noise estimation model *μ*_*θ*_ and then generate SF data from a random noise *ϵ* under the condition of cell shape *c* iteratively. When training the model, the noise *ϵ* is generated randomly first, and this noise is used to destroy the original SF data *x*_0_. Furthermore, the destroyed SF data are used to predict the noise *μ*_*θ*_(*x*_*t*_, *c*, *t*), and finally, the predicted noise is expected to be similar to the actual noise *ϵ*. The size of the input Gaussian noise *ϵ* and cell contour images *c* along with the output SF images *x*_*t*_ are 256 × 256. The total length of timestep iteration *T* is 800, and the linear method with *τ*_*i*_ = ⌊0.1*i*⌋ is utilized to sample the sub-sequence and the length is *S* = 80. For the variance schedule method, we use the cosine schedule [45] to control ${\left\{\alpha \right\}}_{t=1}^{T}$. After the 1500 epochs training with the loss function convergence, the network can generate SF data from random noise according to the input cell shape by gradually subtracting noise based on timesteps. We have a SF dataset comprising 1293 microscopic images. To evaluate our machine learning system, we conducted 13 separate training and testing cycles. In each of the first 12 cycles, we randomly selected 1193 images for training and a different set of 100 images for testing, ensuring no overlap between training and testing sets across cycles. For the final 13th cycle, due to the limited number of remaining images, 1200 images were used for training and the last 93 for testing. During each training phase, the selected images processed with random rotations and flips to enhance the robustness of our system. This approach allowed us to assess the accuracy of the machine learning system across all 1293 images. We use the training parameters as follows: batch size = 16, learning rate for the Adam optimizer is *ε* = 1.5 × 10^−4^. The whole progress is realized with the PyTorch framework. Nvidia RTX A4000 and Nvidia Titan RTX accelerate the whole learning process.

### Quantification of geometric features: SFs and cell morphology

In this subsection, we explain the method to quantify the cell morphology (area, principal direction, aspect ratio, circularity) and the geometry of the actin SFs (total length, principal direction, alignment). The SFs are extracted using our CNN model, detailed in Step C of the previous section. The segmentation results are further elucidated in the subsequent section. We commence by defining the total length and the principal direction of SFs as follows:

$$\begin{array}{c} \begin{array}{c} \ell =\sum_{m}^{M}\ell_{m}\;\bar{\phi }=\sum_{m}^{M}\phi_{m}\cdot \omega_{m} \end{array} \end{array}$$ (4)

where *l*_*m*_ is the length of *m*-th single SFs, *M* is the total number of the SFs in an individual cell, *ϕ*_*m*_ is the angle of *m*-th single stress fiber, *ω*_*m*_ = *l*_*m*_/*l* is the weight function. To evaluate each *l*_*m*_ and *ϕ*_*m*_ in every segmented SF that is image processed by CNN, we apply an edge detection method LSD (Line Segment Detector) [46]. This method allows us to extract straight lines with corresponding two endpoints from the images, and can be used to compute the length and the direction of each SF. To quantify this SF alignment, we utilize the order parameter *S* [47] defined as

$$\begin{array}{c} S=\sum_{m}^{M}\left[2\;{\text{cos}}^{2}\left(\phi_{m}-\bar{\phi }\right)-1\right]\cdot \omega_{m}. \end{array}$$ (5)

The parameter is *S* = 1 when there is a perfect alignment in a certain orientation, while it is *S* = 0 for random orientations.

Also, we examine the relationship between the principal orientations of cells and SFs, quantified as $\left|\bar{\phi }-\psi \right|$. The cell direction, *ψ*, is determined by the angle between the x-axis and the major axis of an ellipse, which has the same second moments as the cell outline. This can be expressed mathematically as:

$$\begin{array}{c} \psi =\text{arctan}(\frac{2\mu_{11}}{\mu_{20}-\mu_{02}}). \end{array}$$ (6)

The second central moments *μ*_20_, *μ*_02_, and *μ*_11_ are defined as:

$$\begin{array}{c} \mu_{20}=\sum_{x,y}{\left(x-\bar{x}\right)}^{2}f\left(x,y\right)\;\mu_{02}=\sum_{x,y}{\left(y-\bar{y}\right)}^{2}f\left(x,y\right)\;\mu_{11}=\sum_{x,y}\left(x-\bar{x}\right)\left(y-\bar{y}\right)f\left(x,y\right) \end{array}$$ (7)

where *f*(*x*, *y*) represents the intensity of the pixel at coordinates (*x*, *y*), and $\left(\bar{x},\bar{y}\right)$ is the centroid of the cell outline region.

Subsequently, it has been well known that the orientation of SFs is closely related to the cell direction [48, 49], thus the relationship between cell area and the total length of SFs can serve as one of the measurements. Note that the cell area is evaluated by counting the pixel number surrounded by the cell contour.

## Results and discussion

### Accuracy of the SF segmentation using CNN

First, in order to ensure the accuracy of training data, we evaluate the segmentation performance using our CNN method. The ground truth data (*N*_test_ = 10) are produced by manually tracing the SFs with freehand lines, and pixels with SF are set to True (1) while the backgrounds are set to False (0). We compared the segmentation performance of two different methods: our CNN method and a filament sensor [50], which is an image analysis based SF segmentation method. The output images of the CNN are in grayscale (0–255) and they are binarized with a threshold of 128 for the segmentation, while the filament sensor provides the extracted fiber information with the endpoint-based data. For a pixel-wise comparison, we first convert the endpoint-based data into images by connecting the endpoints with 1-pixel wide lines. Second, we introduced a kernel operation (mask size: 8 × 8 (1.1*μ*m × 1.1*μ*m)) to convert the 1-pixel width into a typical SF width (∼1*μ*m). The kernel slides across each pixel, and when an SF pixel is detected within the mask, all pixels in the current 8 × 8 area covered by the mask are set to True (SF region). Note that we also applied this mask operation to our binarized images of CNN for a fair comparison. Note that the comparison is done under the resolution of 1024 × 1024.

**Fig 4:**
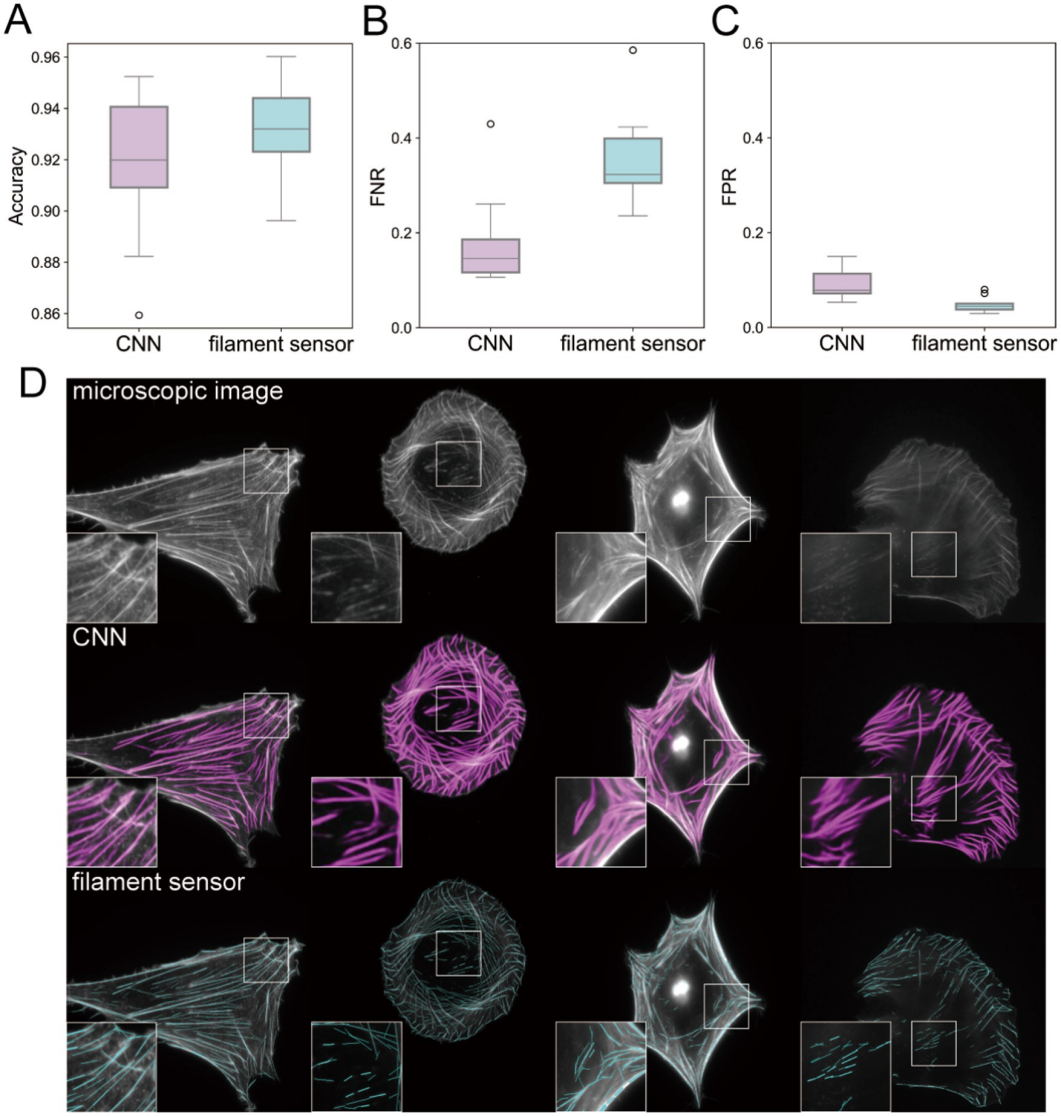
SF segmentation accuracy using CNN method. (A) Accuracy comparison of SF segmentation results using our CNN method and the filament sensor. The box plots describe the minimum, first quartile, median, third quartile, and maximum. (B) False negative rate (FNR) and (C) False positive rate (FPR) of SF segmentation results using our CNN method and the filament sensor, with lower values indicating better performance. (D) Visualization of SF segmentation results using our CNN method and the filament sensor.

The accuracy of our CNN method and the filament sensor is shown in Fig 4A. The accuracy is calculated as the ratio of true positive (TP) and true negative (TN) pixels to the total number of pixels. The results show that our CNN method has a similar performance (0.917 ± 0.027) compared to the filament sensor (0.932 ± 0.017). For further comparison, we also compute the false negative rate (FNR) and false positive rate (FPR) as follows: FNR = FN/(TP + FN) and FPR = FP/(FP + TN), where FP and FN represent the number of pixels of false positive and false negative, respectively. Fig 4B and 4C show that our CNN method has a lower FNR (0.179 ± 0.095) and slightly higher FPR (0.090 ± 0.032) compared to the filament sensor (0.351 ± 0.096) and (0.047 ± 0.016), respectively. This indicates that our method is better at capturing fine details in local SFs as seen in Fig 4D.

In conclusion, the filament sensor achieves accuracy that is comparable to machine learning, even if the approach is purely based on image processing techniques. One small negative aspect of this method is that it requires artificial adjustment of hyper-parameters during the pre-processes and binarization processes, which could lead to time-consuming parameter tuning. Although these two methods have similar performance, we choose to use our image augmentation method and the CNN approach, and we used these segmented SF images to train our diffusion model.

### Prediction of SF localization using diffusion model

**Fig 5:**
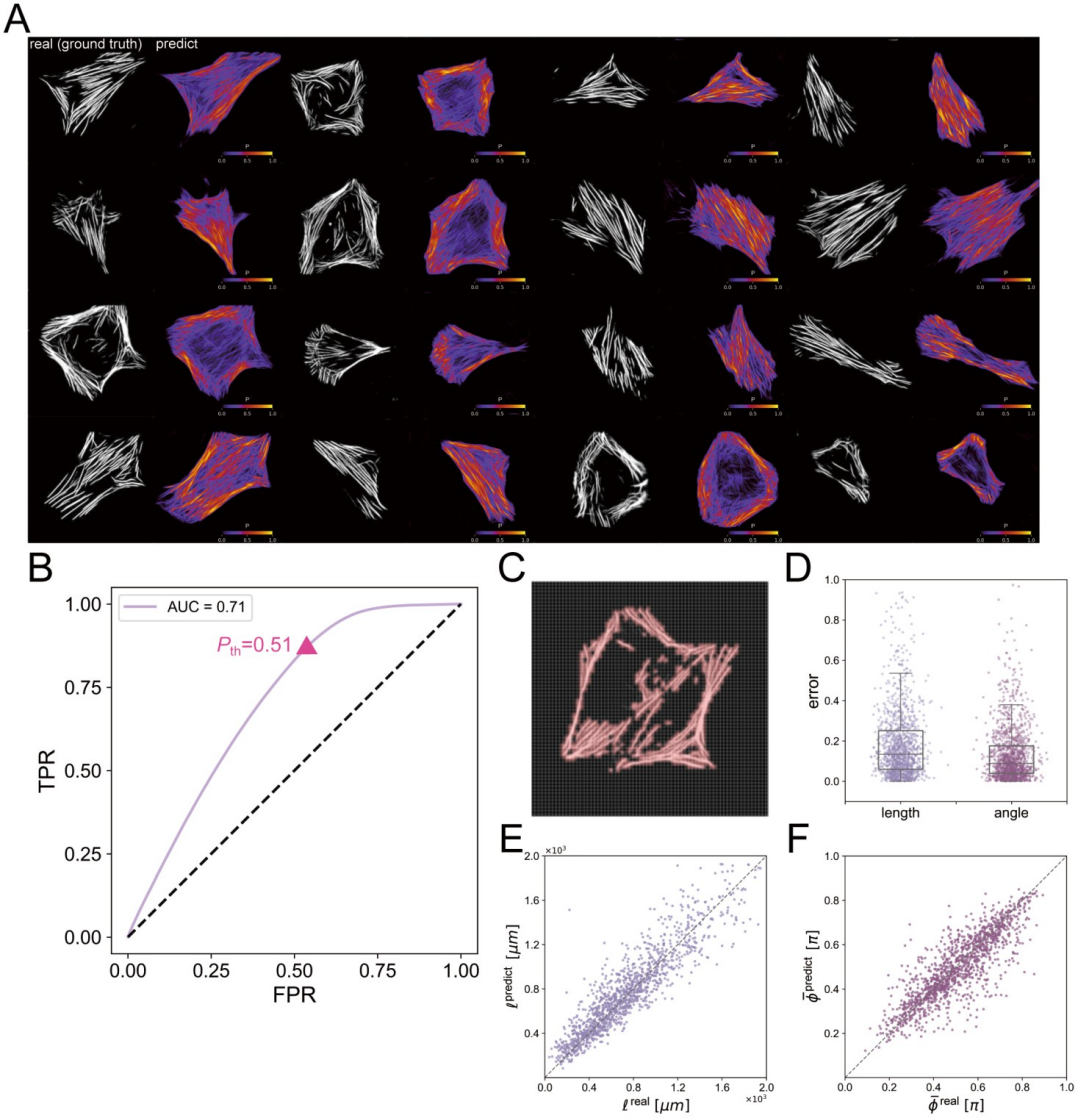
Predicted SFs based on the diffusion model. (A) Comparison of real (ground truth) SF images and predicted SF region from the diffusion model. The contour *P* shows the probability of the SF localization. (B) ROC curve and the corresponding AUC score for each threshold *P*_th_ value. The ROC curve illustrates the FPR (false positive rate) versus the TPR (true positive rate) of the prediction. (C) A sample image explaining the data preparation for the ROC analysis. The image is separated into 64 × 64 grids, and each grid is categorized as the SF region (pink) if any of the pixels inside the grid are pixels with SF (white). (D) Error of predicted SF length *ℓ*^redict^ and direction ${\bar{\phi }}^{\text{predict}}$ from the diffusion model. (E) Comparison of ground truth SF length *ℓ*^real^ and predicted SF length *l*^predict^, with the dashed line indicating equal values. (F) Comparison of ground truth SF direction ${\bar{\phi }}^{\text{real}}$ and predicted SF direction ${\bar{\phi }}^{\text{predict}}$, with the dashed line indicating equal values.

We now employ our conditional diffusion model to generate SFs from the input cell outline images and evaluate the performance of this approach. Since the results of the diffusion model depend on both input cell shape and Gaussian noise, we generate 100 SF images from one input cell outline and calculate the average distribution field of all the generated 100 SF images. Note that each output image from the diffusion model is in grayscale (0–255), and the image is binarized with a threshold of 128 to separate SF and non-SF regions. We define *P* ∈ [0, 1] as the probability that a pixel is considered as SFs; if a pixel is considered as the region of SFs for *N*_S_ images out of *N*_I_ generated images, the probability is *P* = *N*_S_/*N*_I_. The spatial distribution of *P* is displayed in Fig 5A, and the figure shows that the distribution of SFs predicted by the diffusion model exhibits good agreement with the real data. In the appendix, the results before aggregating into the average distribution are illustrated in Fig A in S1 Text.

Next, we quantitatively evaluate the accuracy of the prediction by comparing the extracted SFs positions with the real SFs. In order to evaluate the overlap between predicted/real SFs positions, we first divide the images into 64 × 64 grids as shown in Fig 5C. Each grid size is 2.2*μ*m × 2.2*μ*m, and we set this resolution since the size is suitable for capturing SFs that have a finite size with a typical width ≤ 1.0*μ*m [11]. Second, each grid is labeled as SF or non-SF regions for both predicted/real images: the grid is labeled as SF region if more than one pixel inside the grid is categorized as SF. For the real images, the SF segmentation method introduced in the previous subsection is utilized to extract the pixels with SFs. For the predicted images, we binarize the spatial probability with a threshold *P*_th_ and define SF containing pixels with a criteria *P* > *P*_th_. Based on these grid labels, we finally evaluate the performance using the ROC (Receiver Operating Characteristic) curve and the AUC (Area Under the Curve) as shown in Fig 5B. Note that we excluded the extracellular area and used the inner area of the cell for the ROC analysis. As shown in Fig 5B, the ROC curve is generated based on FPR (false positive rate) and TPR (true positive rate) values for different *P*_th_ and the performance reached the maximum at *P*_th_ = 0.51: note that we judge the best performance by the distance from the ideal performance (FPR, TPR) = (0.0, 1.0). In Fig B in S1 Text, we also evaluate how the performance differs by changing the number of averaged images *N*_I_. We found the prediction is not accurate if we only use a single image (AUC score 0.55 for *N*_I_ = 1) while the performance converges to 0.71 for *N*_I_ = 100. From this analysis, it can be inferred that a single generated image represents just one possible distribution of SFs under the constraint of a cell shape, and we can obtain reliable positions only after averaging multiple images generated from various noise inputs.

In order to further evaluate whether the predicted SFs configurations have similar properties as the real ones, we now extract the fiber geometry by applying an edge detection method LSD (line segment detector) [46] to both predicted/real images. We first compare the generated 1293 cells with the ground truth by measuring the total length *ℓ* and the principal direction $\bar{\phi }$. The length and angle are calculated as Eq (4), and the error is defined as |*ℓ*^predict^ − *ℓ*^real^|/*ℓ*^real^ and $|{\bar{\phi }}^{\text{predict}}-{\bar{\phi }}^{\text{real}}|/{\bar{\phi }}^{\text{real}}$ where superscripts predict and real indicate the value of the prediction or the ground truth. Note that *ℓ*^redict^ and ${\bar{\phi }}^{\text{predict}}$ are obtained from the average *P* of the generated 100 SF images. As shown in Fig 5D, the error of the predicted SF length is 21.1 ± 31.9 [%], and the principal direction is 13.6 ± 15.2 [%]. Also, Fig 5E and 5F show that the length *ℓ* and the principal direction $\bar{\phi }$ are highly correlated with those of the ground truth. Subsequently, upon conducting the Student’s T-test to compare the predicted SF parameters with those from the ground truth data, we obtained p-values of 0.09 for angle, 0.106 for length, and 0.287 for the order parameter. These p-values, all above the common significance threshold of 0.05, suggest that there are no statistically significant differences between the ground truth and predicted parameters.

**Fig 6:**
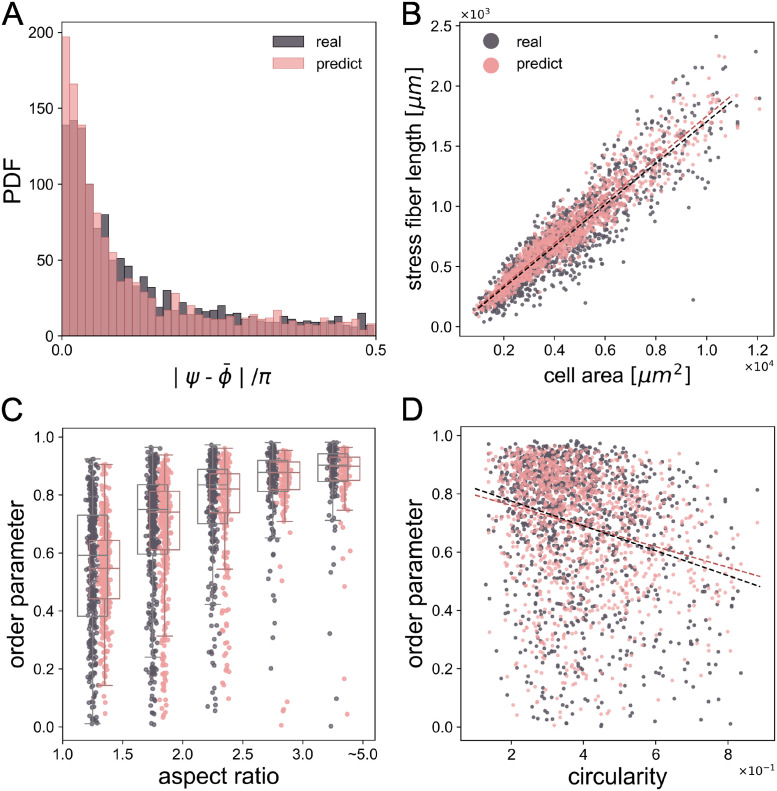
Quantitative analysis of the relation between predicted SF dynamics and cell morphologies. (A) Probability distribution function of the angle differences between cell direction *ψ* and SF direction $\bar{\phi }$ in both ground truth data and predicted data from the diffusion model. (B) Relationship between SF length and cell area in both ground truth data and predicted data. (C) Correlation between ground truth SF order parameter *S* and predicted SF order parameter *S* from the diffusion model, and cell aspect ratio, divided into 5 intervals. The box plots describe the minimum, first quartile, median, third quartile and maximum. (D) Correlation between cell circularity and SF order parameter in both ground truth data and predicted data.

Additionally, by utilizing the quantitative measurement with Eqs (4)–(6), we explored the correlation between the generated SFs and the actual SFs with the cell morphology, and confirmed whether this relationship remained the same as the actual data. Fig 6A demonstrates the cell direction *ψ* and the SF direction $\bar{\phi }$ are indeed parallel most of the time. The figure also shows that the prediction has good agreement with the actual distribution. Fig 6B indicates that the total length of SFs increases linearly with the cell area. Although it has been reported that the length of SFs is longer for cells with larger area [51, 52], this is the first report, to the best of the authors’ knowledge, that shows a linear relationship between the length of SFs and the cell area, and this relationship can be fitted with a linear function with a slope 0.172 (*ℓ* = 0.172*A* − 20, where *A* is the cell area). Note that this linear relationship *ℓ* ∝ *A* could be understood by assuming that the area of SFs *A*_SF_ relative to the cell area *A* is nearly a constant. When the area ratio Φ = *A*_SF_/*A* is a constant, the area of SFs can be rewritten as *A*_SF_ = *A*Φ = *wℓ* where *w* is the typical width of the SFs. This simple model indicates that the cell area *A* = *wℓ*/Φ is proportional to the total length *ℓ*. Note the ratio was Φ = 0.10 ± 0.02 for our cells.

Next, we evenly divided all the SF data into 5 groups according to the cell aspect ratio from 1.0 to 5.0. As shown in Fig 6C, the average values of order parameter *S* for each interval are 0.548, 0.736, 0.806, 0.869 and 0.899, respectively. The current result is in agreement with previous studies that have shown that the alignment of SFs tends to increase with the aspect ratio of the micro-patterns that constrain the cell shape [17, 18, 20], or the alignment of actin filaments in microchambers [19]. This illustrates that elongating cells tend to have a higher alignment of the SFs. Meanwhile, as the aspect ratio increases, the IQR (interquartile range) range of the order parameter *S* tends to decrease, demonstrating that the alignments of SFs in a cluster of cells tend to converge as the cell aspect ratio increases. As for the predicted results coming from our diffusion model, the average value of order parameter *S* in Fig 6C for each interval is 0.548, 0.720, 0.804, 0.868, 0.899, which shows good agreement with the actual one.

Subsequently, we found no significant correlation between cell area and order parameter *S* (*R* = −0.032), and the cell circularity has a weak correlation with *S* (*R* = −0.272) as shown in Fig 6D, which means the order parameter decreases with the circularity increases. The same conclusion can be drawn from the predicted SF data, as the order parameter from the predicted SF data also correlates with the cell circularity (*R* = −0.242) as shown in Fig 6D. There is no correlation between cell area and predicted SF order parameter (*R* = 0.020), which agrees with the real SF data.

As demonstrated above, the topology of SFs is highly correlated with the low-dimensional expression of cell morphology, especially with the cell aspect ratio, and our diffusion model proved this by successfully predicting the localization and distributions of actin SFs from the input cell shape images with limited errors. Additionally, the Supplementary S1 and S2 videos further show the application of the proposed system in providing high throughout, real-time prediction of the SF localization during dynamic cell locomotion.

### Effect of curvature in cellular contours

Previous studies have reported correlations between the cell shapes, especially the peripheral arcs, and the intensity of SFs. In this section, we examine whether our machine learning model captures these relationships.

**Fig 7:**
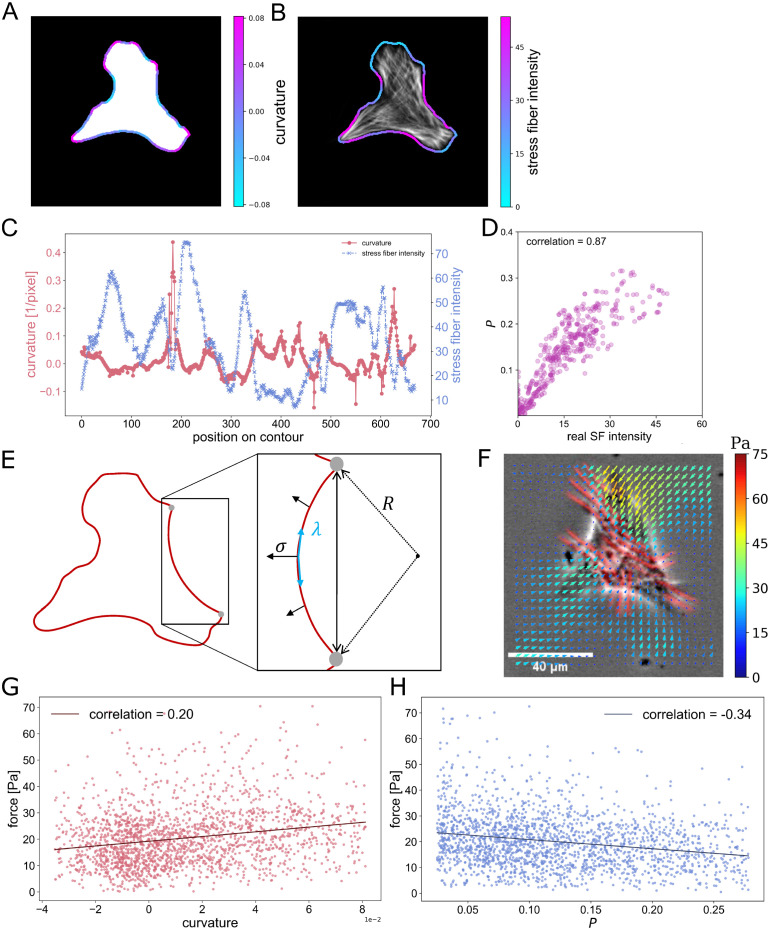
Correlation between local cell curvature, SF intensity and cell contractile force. (A) Visualization of the local curvature values along the cell contour. (B) Visualization of local SF intensity on cell contour. (C) A negative correlation between cell curvature value and SF intensity for a single cell contour. (D) A correlation between the SF intensity and the SF probability *P*. The correlation 0.87 is calucated from 1293 cells, while 500 points are randamly ploted for a clear visualization. (E) Schematic representation of the cell from (A), depicting line tension λ and surface tension *σ*, with their relationship determined by the curvature radius *R*. (F) The contractile force measured using the WFM method. (G) Positive correlation between curvature and force, and (H) negative correlation between the SF probability *P* and force. The correlations were calculated for each point on the cell contour (360 images). For a clear visualization, 2000 points are randomly selected.

From the microscope images, we firstly evaluate the relation between the local curvature of the cell contour (Fig 7A) and the SF intensity along the cell contour *I*_SF_ (Fig 7B). The local curvature of the cell contour is obtained by fitting the contour inside a circular mask with diameter *L* with a quadratic function *g*(*x*) = *ax*^2^ + *bx* + *c*, where *L* = *P*_cell_/30 is one-thirtyth of the cell perimeter *P*_cell_. Note that the curvature is evaluated as

$$\begin{array}{c} \kappa =\frac{\left|g\prime \prime \left(x\right)\right|}{{\left(1+{\left(g\prime \left(x\right)\right)}^{2}\right)}^{\frac{2}{3}}} \end{array}$$ (8)

and the positive curvature corresponds to a convex arc while the negative curvature is a concave arc. Simultaneously, the SF intensity *I*_SF_ is quantified by averaging the pixel intensity inside the circular mask. Note that we only use the intensity within the cell for the averaging, and the intensity outside is ignored. By plotting these parameters of a single cell contour as illustrated in Fig 7C, there is a tendency for the curvature to have an opposite trend from the changes in the SF intensity. The correlations between these two quantities indeed have negative values (*ρ* = −0.31 ± 0.20; 1293 images), and this tendency is in agreement with the previous reports [22, 53–55]. Secondly, we use the results from the diffusion model. Same as the averaging process of the curvature, we obtain the averaged probability *P* along the cell contour. As shown in Fig 7D, surprisingly, there is a high positive correlation *ρ* = 0.87 between the experimentally obtained *I*_SF_ and the SF probability *P*. The probability *P* also has the same negative correlation (*ρ* = −0.42 ± 0.19; 1293 images) with the local curvature. From this analysis, it is suggested that the SF probability *P* from the diffusion model not only provides the SF distribution but also the local intensity or density of the SFs.

The mechanical relationship between the SF intensity *I*_SF_ and the curvature *κ* can be understood by the Laplace law [22], which describes the force balance between the contractile stress *σ* and the line tension λ and at the concave arcs as shown in Fig 7E. Note that both *σ* and λ originate from the contraction of the actin cortex or the surface tension of the plasma membrane [22]. By substituting Hooke’s law λ = *EAε* to Laplace law *R* = λ/*σ*, we obtain

$$\begin{array}{c} R=\frac{1}{\kappa }=\frac{EA}{\sigma }\varepsilon \end{array}$$ (9)

where *R* is the curvature radius, *κ* is the curvature, *E* is Young’s modulus of SFs, *A* is the cross-sectional area of SFs and *ε* is the strain of SFs. This equation tells that the curvature is determined by the balance between the elasticity *EAε* and the cell contraction *σ*. SFs with large intensity *I*_SF_ are expected to have large rigidity *EA* [22]. The negative correlation between *I*_SF_ and *κ* can be explained by Eq (9) as large *EA* results in smaller curvatures *κ*.

Given that the curvature and SF intensity, the latter of which serves as an indicator of rigidity *EA*, are the measurable variables, our system may be possible to predict the cellular contractile stress with a certain accuracy. Next, to quantify the relationship between the local curvature and the contractile force, we further conducted an analysis using data from our previous studies [56, 57]. As described in our previous studies, we cultured A7r5 cells on PDMS substrates that can visualize the contractile force with wrinkle patterns of underlying PDMS [56], and our wrinkle force microscopy (WFM) technique [57] allows us to predict the contractile force from the wrinkle pattern as shown in Fig 7F and supplementary S3 video. The total number of the captured cell images is 360, and during the experiment, we obtained the shape of the cells and the deformation of the substrate surface, representing changes in cellular contractility. To evaluate the local force at each contour point, we averaged the contractile force that is closer than the length *L*.

Fig 7G shows that the force has a positive correlation with the curvature, suggesting that the force is stronger for contours that have larger convex (*κ* > 0). This tendency can be explained by the fact that the focal adhesions, which are the force transmission sites, tend to be located at the convex region because of their nature as the anchoring points [58, 59]. Fig 7H shows that the SF probability *P* has a negative correlation with force, and it suggests that the probability *P* from our diffusion model can be a good candidate to roughly estimate the magnitude of the contractile forces. This negative correlation between the force and *P* can be explained by the two correlations: the positive correlation in force and *κ* (Fig 7G) and the negative correlation in *κ* and *P*. Together with the force balance given in Eq (9), the probability *P* might be a potential indicator to estimate the cellular force [60] in the future.

### Virtual experiment: From cell shape to SFs

In previous sections, we successfully predicted the SF localization from the cell shapes using a diffusion model-based machine learning system and evaluated the accuracy. From a different perspective, it can be said that our system now “installed” the geometric relation between the cell shape and SF localization based on the big data from the experiments; in other words, the system could be used to generate the SFs geometry not only from the real cells but even from artificial cell shapes. Although one wants to extract principles that underlie between two different parameters, such as the aspect ratio of cells vs SF localization, it is not trivial to observe cells under ideal situations in experiments. In this section, we show that this system can be utilized to simulate the SF localization for idealized virtual cell shapes. We term this SF generation based on the virtual shape as a “virtual experiment”, and show that this system would be a tool to predict hidden principles of the cell geometry.

**Fig 8:**
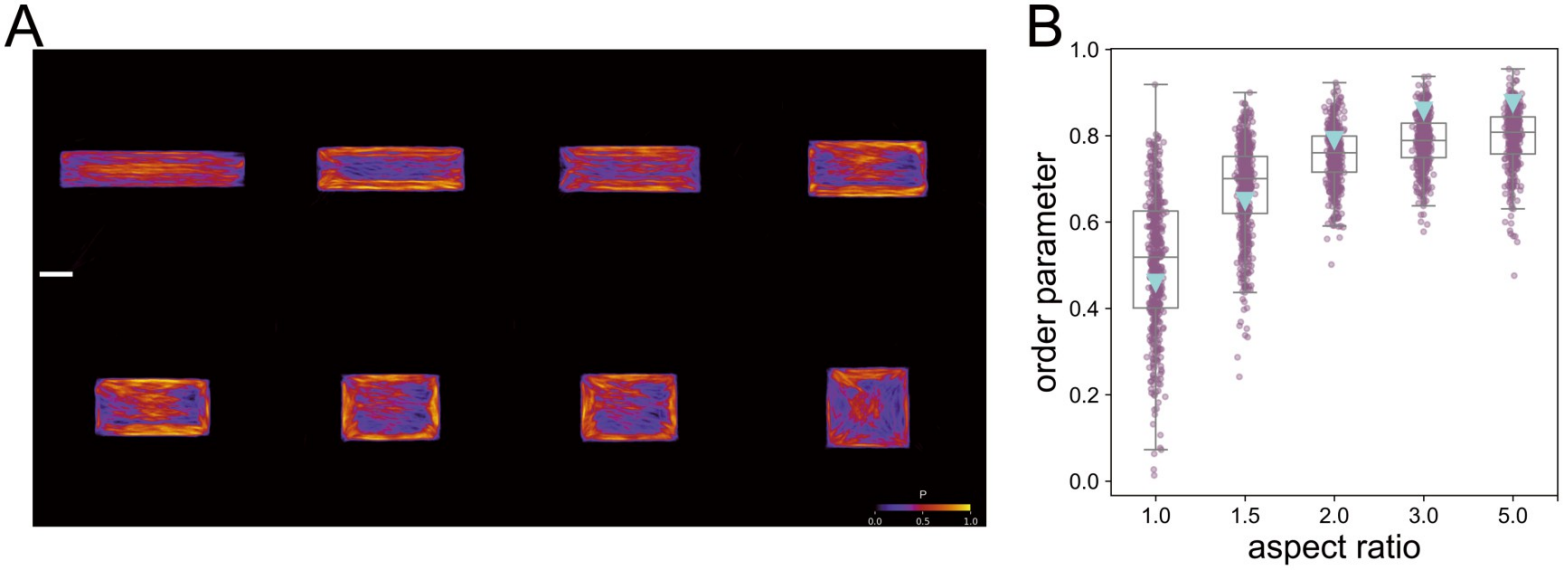
Results from generated SFs comparing with previous studies. (A) Probability *P* of SFs under rectangular constraints predicted by the diffusion model, and a scale bar is 20 *μ*m. (B) Relationship between simulated SF order parameter and constraint area aspect ratio, compared with results from a previous study (marked as ▽) [19]. The box plots describe the minimum, first quartile, median, third quartile and maximum.

First, we generate SFs in this virtual experiment with the constrained cellular contour. This region in our virtual experiment is defined as a rectangle with aspect ratios varying from 1.0 to 5.0, and an area limited to 2.50 × 10^3^ *μ*m^2^. We then create 100 SF images and calculate the average SF distribution, as illustrated in Fig 8A. The order parameter for each constraint is presented in Fig 8B, and the figure indicates a continuous increase with the aspect ratio. In the previous study [19], actin filaments were confined within several rectangular microchambers, each 100*μ*m long, with varying aspect ratios adjusted by changing the chambers’ areas. The order parameter from this experiment, marked as ▽ in Fig 8B, demonstrates a trend that aligns with our findings. This comparison shows the similarity between the trends observed in the physical experiment and our simulated results, indicating the effectiveness of our virtual experiment approach.

**Fig 9:**
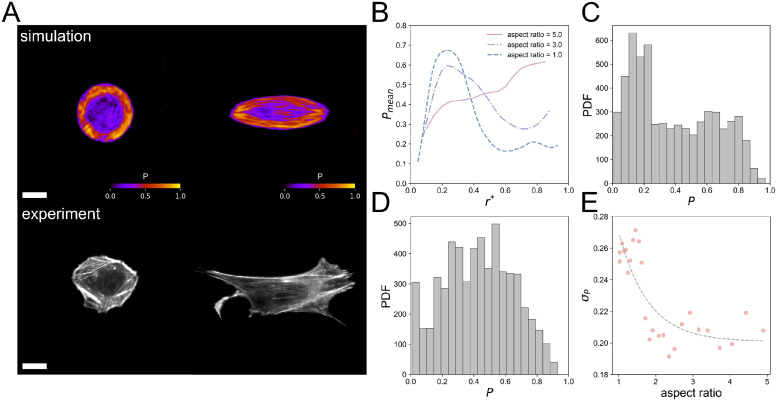
Results from the virtual experiment using the diffusion model. (A) Comparison of generated SFs with geometrical constraints of aspect ratio (*AR* = 1 and 3) to experimental results of HFF-1 cells with comparable geometry constraints. The cells in the experimental group have aspect ratios of 1.1 and 2.8, and areas of 1.83 × 10^3^ *μ*m^2^ and 1.88 × 10^3^ *μ*m^2^, which are similar to the morphology conditions during the virtual experiments. Scale bars represent 20 *μ*m. (B) Relationship between distance from the geometrically constrained edge r* and probability of generating SFs *P*_*mean*_ for three cases of aspect ratio (*AR* = 1, 3 and 5). (C,D) Probability distribution function (PDF) of the probability of generating SFs *P* with a constraint of (C) *AR* = 1 and (D) *AR* = 3. (E) Standard deviation *σ*_*P*_ of *P* decreases as constraint aspect ratio increases.

Second, we attempt to quantify the geometrical relationship between SFs and the corresponding virtual morphologies. We prepare elliptical shapes with aspect ratios *AR* varying continuously from *AR* = 1 to 5 as the input virtual shape, while the area is set constant 1.96 × 10^2^ *μ*m^2^. The generated SFs and the SFs from our experiments are shown in Fig 9A. In the figure, we show generated SFs for two different aspect ratios (*AR* = 1 and 3), and two cells from our experiments that have relatively the same shapes (*AR* = 1.1 and 2.8). Fig 9B shows the average probability *P*_mean_ at the position r*, where r* = *r*/*R*_max_ is the normalized distance from the cell edge, *r* is the actual distance (pixels) from the cell centroid to the cell edge, and *R*_max_ denotes the length of the semi-minor axis of the ellipse. Note that *P* reflects the likelihood of SF formation at each pixel, which is calculated by averaging the distribution of SFs over 100 generated SF images generated from the diffusion model as described in the previous section, and *P*_mean_ is the average value of *P* at locations with the same r*. As also seen in Fig 9B, the circular cell has a high probability *P*_mean_ ∼ 0.7 at the cell edge while the value is small *P*_mean_ ∼ 0.2 at the center. The profile of probability *P*_mean_ becomes flat for cells with larger AR, and this indicates that SF homogeneously distribute inside the cells for high AR. Additionally, we visualize the PDF of the probability *P* as shown in Fig 9C (*AR* = 1) and D (*AR* = 3). The PDF of the probability *P* has a single peak at *P* ∼ 0.5 for elongated cell *AR* = 3. On the other hand, the circular cell has a bimodal profile of *P* and the result again suggests that there is a localization of high/low *P* distribution. In order to quantify this trend, we finally show the standard deviation *σ*_*P*_ of the probability *P* in Fig 9E. While the value *σ*_*P*_ is large for small AR since there are bimodalities in the profile, it gradually decreases with AR since the probability *P* becomes more homogeneous inside the cell. Fig 9E shows that cells with *AR* < 2 would show strong localization in the SF distribution.

In this final section, we performed virtual experiments using the conversion principles that are extracted from our experiments. The generated SFs from the virtual cell shape agree with the previous study of actin filaments. As summarized in the introduction, it is important to understand the geometric relation between cell shapes and cytoskeletons. This conversion will be a powerful supporting tool to find hidden trends as the system can extract averaged geometric features from the big data of experiments. For instance, we found that SFs tend to locate at the cell edge for *AR* < 2 while the probability *P* is expected to be more homogeneous for cells with large aspect ratios. This concept of “installing” the underlying rules can be used not only for this specific task, but can also be extended to other conversions to simulate the characters of cell nature.

## Conclusion

In summary, we proposed a diffusion model-based machine learning system, which allows for predicting the geometries and localizations of SFs from cell morphologies. First, we extract SFs and cell shapes from raw fluorescence microscopic images using CNN and image processing methods. From the extracted SFs, we found the total length of SFs in a single cell linearly increases with the cell area. We also found that SFs exhibit an inherent tendency to align in identical directions upon increased cell aspect ratio. Note that we managed to draw these conclusions by extracting averaged geometric features from big data, with the help of machine learning. Second, we train the system with the diffusion model using two corresponding image sets, SF images and cell shape images, as training data. The predicted SFs are highly correlated with our experimental data and show the maximum AUC of 0.71. The errors were 21.1 ± 31.9 [%] for SF length and 13.6 ± 15.2 [%] for SF principal direction. Finally, we show that our system can be utilized for virtual experiments to predict the localization of SFs from artificial cell shapes as inputs. From the virtual experiment, we found that SFs of cells with small aspect ratios tend to localize at the cell edge while cells with large aspect ratios have homogenous distributions of SFs.

Cell biology has long examined the connection between cellular morphology and function [61] and has studied individual cells in detail [9, 11, 62]. The ability of cells to contract, which is largely driven by SFs, particularly in mesenchymal cell types, has been linked to a range of processes such as proliferation, differentiation, apoptosis, and tumorigenesis. Cell shape is one of the most common features altered upon cellular processes, including epithelial-mesenchymal transition [9, 63] and can be obtained relatively easily through optical microscopes, and at the same time, actin SFs are vital components of cells in controlling many cellular functions, with effects ranging from migration and proliferation to apoptosis. The high accuracy of the prediction of the spatial distribution of SFs is achieved based on data of endogenous, but not exogenous, actin filaments, suggesting that this conversion from cell geometry to SFs serves as a powerful system. Specifically, this approach provides a quantitative subcellular map of the endogenous actin filaments even without allocating a specific fluorescence wavelength for imaging. The circumvention of using one wavelength makes the experiment significantly easier because, while theoretically, multi-wavelength imaging is possible and has been extensively performed, some experimental procedures, such as double transfection of exogenous molecules, reduce the efficiency of the experiment and integrity and intactness of the cells. Although the prediction error of our current analysis might be big in realizing the full concept, this method could become a valuable tool with further refinement and optimization for subcellular imaging and analysis in the future.

Our system potentially enables finding further hidden geometric principles regarding how the configuration of subcellular structures, in addition to SFs investigated here, is determined by the cell boundary; e.g., some molecules such as eEF2 are known to be determined in their subcellular position according to the distribution of SFs [11]. By combining with our previous study [56, 57], which is a machine learning based approach that can extract cellular force distributions from microscope images, we analyzed how cell morphologies are connected with cell mechanics including its potential to generate physical forces. Our approach provides a powerful framework to understand the geometric features of cells, which would support new findings in the field of cell biology and mechanobiology.

## Supporting information

S1 Text Supporting materials. Supporting materials for “Appendix A. SF generation results using diffusion model” and “Appendix B. Analysis of AUC score for SF prediction”.
**Figure A in S1. Text Detailed View of SF Generation Using the Diffusion Model**.
**Figure B in S1 Text Analysis of the AUC score.** (A). The AUC score increases with the increase of averaged generated SF images *N*_I_. (B). The PDF and CDF analysis for the AUC score between SFs in generated (*N*_I_ = 100) and ground truth images demonstrates that 90% of our generated sample can have a score from 0.62 to 0.85.
(PDF)

S1 Video Prediction of SF positions from a time-lapsed movie of cell geometry. Extract HFF-1 cell shapes from live videos and generate corresponding SF data using a trained diffusion model.
(AVI)

S2 Video Prediction of SF positions from a time-lapsed movie of cell geometry. Same as S1 Video. (AVI)

S3 Video Combining predicted SF data with WFM method. The video showing (left) predicted SF distribution and (right) the cellular contractile forces using WFM (Wrinkle Force Microscopy), of a A7r5 cell.
(AVI)
