# Supplementary material for: Diffusion model predicts the geometry of actin cytoskeleton from cell morphology
Source: PLoS Comput Biol. 2024 Aug 5;20(8):e1012312. doi: 10.1371/journal.pcbi.1012312 (PMC11326640; doi:10.1371/journal.pcbi.1012312)
Supplement: S1 Text — Supporting materials for “Appendix A. SF generation results using diffusion model” and “Appendix B. Analysis of AUC score for SF prediction”. Figure A in S1. Text Detailed View of SF Generation Using the Diffusion Model. Figure B in S1 Text Analysis of the AUC score. (A). The AUC score increases with the increase of averaged generated SF images NI. (B). The PDF and CDF analysis for the AUC score between SFs in generated (NI = 100) and ground truth images demonstrates that 90% of our generated sample can have a score from 0.62 to 0.85. (PDF) [file pcbi.1012312.s001.pdf]

Appendix of “Diffusion model predicts the geometry of actin cytoskeleton from cell morphology”

Honghan Li, Shiyou Liu, Shinji Deguchi, Daiki Matsunaga.

Supporting information

Appendix A. SF generation results using diffusion model

In this section, we present actual individual images of SFs generated by the diffusion model. As illustrated in Fig A, the arrangement and distribution of SFs in the samples have similar trends under one specific cell geometry, but some areas are different from GT. Note that GT images are SF segmented images using our CNN method (see method section “Step C: Actin SFs segmentation”). These individual images together form a probability map of the SF distribution.

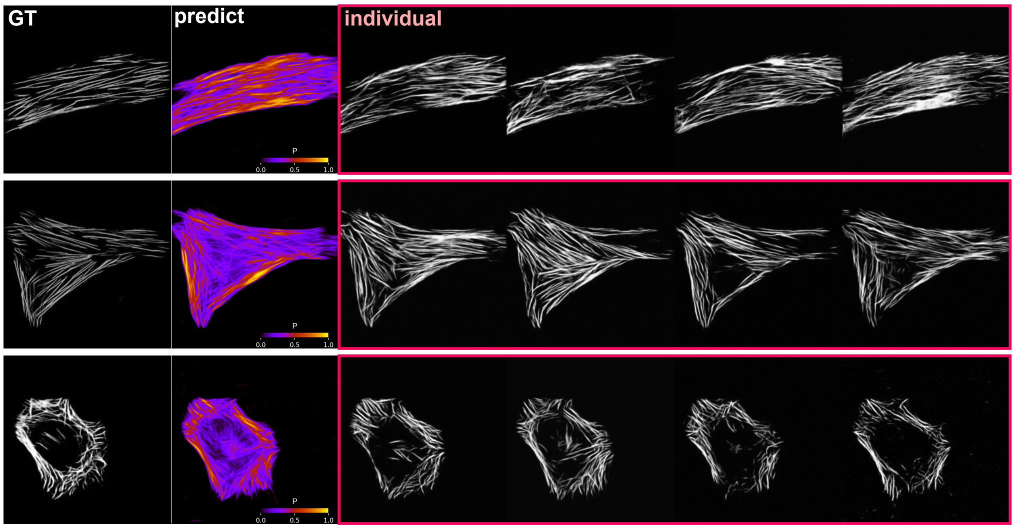

Fig A. Detailed View of SF Generation Using the Diffusion Model.

Appendix B. Analysis of AUC score for SF prediction

In the main text, we generated 100 images and averaged the probability in order to estimate the SF positions as shown in Fig 5. In this appendix, we first test how the accuracy of the prediction differs by the number of generated images. Fig B-I demonstrates that the AUC score progressively increases with more sample generations. Although the performance is low (0.55) at $N_\mathrm{I} = 1$, the score converges to 0.71 by increasing the sample number to $N_\mathrm{I} = 100$.

Second, to further explain Fig 5B in detail, Fig B-II illustrates the probability density function (PDF) and cumulative density function (CDF) for the AUC score at $N_\mathrm{I} = 100$. The PDF shows the likelihood of each score, while the CDF indicates the

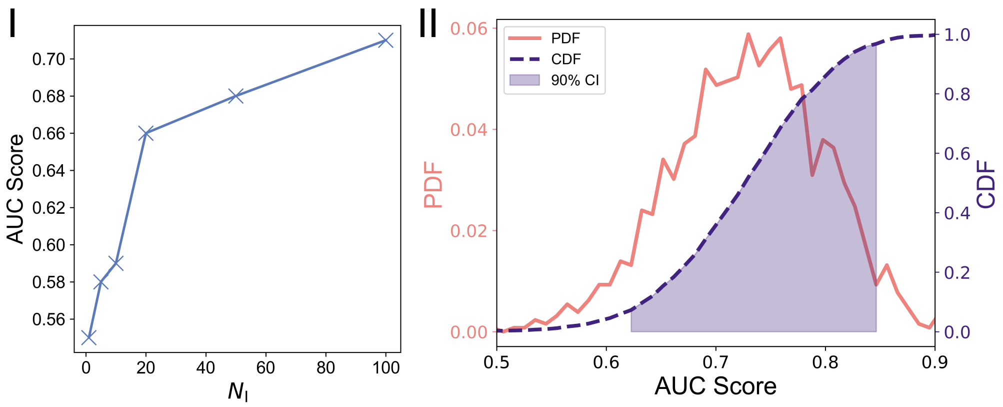

Fig B. Analysis of the AUC score. (I) The AUC score increases with the increase of averaged generated SF images N_I. (II) The PDF and CDF analysis for the AUC score between SFs in generated ($N_\mathrm{I} = 100$) and ground truth images demonstrates that 90% of our generated sample can have a score from 0.62 to 0.85.

cumulative probability up to each score point. The 90% confidence interval (CI) displayed highlights the range within which we can expect 90% of our generated samples to fall to an AUC score ranging from 0.62 to 0.85.
